# Cognitive‐Behavioral Performance Pre‐ and Post‐Surgery in Parkinson’s Disease Patients Undergoing Deep Brain Stimulation

**DOI:** 10.1002/alz.085109

**Published:** 2025-01-03

**Authors:** Maria Cecilia Fernandez, Ximena Giordano, Waleska Berrios, Licina Barbieri, Agustina Ciceri, Gaston Moreno Milicich, Pablo Vera Cornejo, Carla Stefani, Lucia Ciancaglini, Jose Bestoso, Diego BAUSO

**Affiliations:** ^1^ Hospital Italiano de Buenos Aires, Buenos aires Argentina; ^2^ Hospital Italiano de Buenos Aires, Buenos Aires Argentina; ^3^ Churruca Visca Hospital, Buenos Aires Argentina; ^4^ Hospital Italiano Buenos Aires, Buenos Aires Argentina

## Abstract

**Background:**

Parkinson’s Disease (PD) is known to cause cognitive and behavioral problems, especially in executive‐attentional areas. For some patients, medication is not effective, and surgery is considered a treatment option. However, changes in mood and cognition have been reported after surgical intervention in the subthalamic nucleus (STN) or the internal globus pallidus (GPi), but the literature is inconsistent. This study aims to examine PD patients' cognitive and behavioral performance before and after deep brain stimulation (DBS) surgery in a patient population from Argentina.

**Method:**

Our study involved a retrospective analysis of 24 patients who underwent DBS surgery, half of whom received surgery in the STN area and the other half in the GPi. We evaluated cognitive function using neuropsychological tests and neuropsychiatric symptoms using the Beck Depression Inventory and Neuropsychiatric Inventory before and 1 year after the surgery. Variables of age, sex and years of education were evaluated and average values of each domain were calculated in the pre‐surgical test, to be later compared with values obtained in the post‐surgical evaluation, according to the brain nucleus operated on.

**Result:**

The study included patients between the ages of 43 and 69 with an average education level of 10.5 years. After surgery, those who underwent STN intervention (83.3% male) showed improved executive‐attentional domains but a slight decline in visuospatial construction and semantic fluency. Behavioral improvements were observed, with a decrease in symptoms from 12.5% pre‐surgery to 2.33% post‐surgery, while mood worsened (37.5% vs. 41.7%), but not significantly. On the other hand, patients undergoing GPi intervention (83.3% male, 16.7% female) showed slight declines in memory, executive‐attentional functions, and semantic fluency but positive changes in mood‐behavioral aspects (0% and 11.1%, respectively).

**Conclusion:**

We found that patients who underwent DBS experienced subtle changes in cognitive performance a year after surgery. While STN intervention showed improvement, those who underwent GPi intervention experienced a slight, nonsignificant decline. However, the study acknowledges that a larger sample size is needed for more substantial results.

